# A content analysis of thinspiration, fitspiration, and bonespiration imagery on social media

**DOI:** 10.1186/s40337-017-0170-2

**Published:** 2017-09-26

**Authors:** Catherine Victoria Talbot, Jeffrey Gavin, Tommy van Steen, Yvette Morey

**Affiliations:** 1 University of Bath, Claverton Down Road, Bath, BA2 7AY UK; 2 University of the West of England, Coldharbour Lane, Frenchay, Bristol, BS16 1QY UK; 3 University of Exeter Medical School, College House 1.23, St Luke’s Campus, Heavitree Road, Exeter, EX1 2LU UK

**Keywords:** Thinspiration, Fitspiration, Bonespiration, Social media, Body image, Content analysis

## Abstract

**Background:**

On social media, images such as thinspiration, fitspiration, and bonespiration, are shared to inspire certain body ideals. Previous research has demonstrated that exposure to these groups of content is associated with increased body dissatisfaction and decreased self-esteem. It is therefore important that the bodies featured within these groups of content are more fully understood so that effective interventions and preventative measures can be informed, developed, and implemented.

**Method:**

A content analysis was conducted on a sample of body-focussed images with the hashtags thinspiration, fitspiration, and bonespiration from three social media platforms.

**Results:**

The analyses showed that thinspiration and bonespiration content contained more thin and objectified bodies, compared to fitspiration which featured a greater prevalence of muscles and muscular bodies. In addition, bonespiration content contained more bone protrusions and fewer muscles than thinspiration content.

**Conclusions:**

The findings suggest fitspiration may be a less unhealthy type of content; however, a subgroup of imagery was identified which idealised the extremely thin body type and as such this content should also be approached with caution. Future research should utilise qualitative methods to further develop understandings of the body ideals that are constructed within these groups of content and the motivations behind posting this content.

## Plain English summary

Thinspiration refers to imagery commonly shared on social media that encourages a user to be thin. Likewise, fitspiration and bonespiration are imagery that inspire a user to be fit and extremely thin, respectively. There has been limited research that has analysed and compared the physical attributes of thinspiration and fitspiration content, and no research has analysed bonespiration. This study aimed to (i) examine the features of bonespiration content in relation to thinspiration and fitspiration; and (ii) explore how fitspiration compared to thinspiration and bonespiration.

The researchers conducted a content analysis on 734 images with hashtags thinspiration, fitspiration, and bonespiration, shared on the social media platforms Twitter, Instagram, and WeHeartit. Similarities were generally observed between thinspiration and bonespiration; however, bonespiration content contained fewer muscles and more bone protrusions, indicating that bonespiration may represent an exaggerated form of thinspiration. Thinspiration and bonespiration contained more thin, objectified bodies when compared to fitspiration, which contained more muscles and muscular bodies. This indicates that fitspiration may represent a less unhealthy form of content when compared to thinspiration and bonespiration. Fitspiration should still be approached with caution, however, as a small group of fitspiration content was identified that was similar to thinspiration with regards to the presence of extremely thin bodies.

## Background

It has been well established that the mass media has played an important role in communicating beauty ideals [1], namely the thin beauty ideal commonly assigned to femininity. The mass communication of this body ideal may result an unattainable and unrealistic construction of feminine beauty [2, 3]. Exposure to these thin ideals can result in decreased body satisfaction [4–7]. Decreased body satisfaction is a cause for serious concern, especially for adolescent girls as this group commonly experience body dissatisfaction [8]. Indeed, decreased body satisfaction has been associated with unhealthy weight control behaviours and binge eating [9], eating disorder symptomatology [10, 11], depressive symptoms and low self-esteem [12].

Until recently, studies have focussed upon the effects of exposure to conventional mass media, such as newspapers, magazines and television; however, a growing emphasis is now being placed upon the role of social media and the effect it can have upon young people who are consuming and interacting with it [1]. Perloff [1] has discussed the how social media technologies differ from mass media communication, where social media can provide users with a personal outlet, interactivity, a feeling of presence, and a community of like-minded individuals. On social media, users are both sources and receivers of information, who can actively shape online interaction that enhance autonomy, self-efficacy, and personal agency [13].

When social media users go online they are exposed to a multitude of thin-idealised female bodies on their news feed of celebrities, people within their social network, and bodies of people they do not know. It could therefore be argued that social media, with its constant availability for interaction and content creation, has increased the exposure that young women have to certain body ideals [1].

‘Inspirational’ imagery is often shared on social media with the aim of inspiring a user to achieve a certain, often unachievable, body type. Thinspiration refers to content posted on social media that inspires a user to be thin, and this is typically achieved through the presentation of images that contain thin bodies, as well as weight loss quotes and techniques [14]. Thinspiration has received considerable attention within the public eye, where this content has been accused of being dangerous and a contributor towards the onset of eating disorders [15, 16]. This may be because thinspiration has traditionally been associated with the pro-eating disorder (proED) community [17], a community that has identified eating disorders as a lifestyle choice, rather than an illness [18]. More recently, thinspiration content has moved away from its typical association with the proED community and has been shared by everyday social media users [19].

In a content analysis of thinspiration, Ghaznavi and Taylor [19] found that these images tended be sexually suggestive and objectifying, and featured extremely thin, bony, scantily-clad women. Thinspiration, with its emphasis upon the thin body ideal, could therefore be damaging to the body satisfaction of social media users. This is a serious cause for concern, especially if thinspiration content has become part of the everyday social media user’s experience.

After searching the hashtag ‘thinspiration’ on various social media platforms, a user will quickly find co-occurring hashtags that are associated with the proED community, such as ‘#proana’. ‘Bonespiration’ is another commonly featured hashtag and refers to content that idealises the extremely thin body through the presentation of protruding bones [19]. A search for the hashtag ‘bonespo’ on Instagram results in over 130,000 posts, which are being updated daily. Bonespiration has received some antagonism within the public eye [20], but it is generally less well known which could be because this content is still firmly engrained within the pro-ED community. Bonespiration has, therefore, received no attention within the academic literature and as such its relation to thinspiration and the physical characteristics of the bodies featured within this content remains unknown.

By contrast, ‘fitspiration’ has been coined by the fitness community as an allegedly-healthy alternative to thinspiration and bonespiration. Fitspiration encourages a user to achieve a supposedly fit body through images that encourage exercise and healthy nutrition [21]. Fitspiration has been largely used within the public eye to encourage *healthy* bodies [22] and was generally considered to be a positive type of content.

More recently, there has been increasing disdain towards fitspiration, where various studies have suggested that fitspiration may result in increased negative mood, body dissatisfaction and decreased state appearance and self-esteem [23]. The physical characteristics of fitspiration could explain this, as it has been identified that most images contained only one body type: thin and toned [23]. Images were also found to contain some objectifying elements. In an analysis of fitspiration content on Instagram, nearly 90% of the bodies were coded as having low body fat and 55% were coded as muscular [24]. Whilst fitspiration presents itself as a healthy alternative it may, however, contain some negative elements typically found within thinspiration content.

To the authors’ knowledge, only one study to date has directly compared these groups of content. Boepple and Thompson [25] compared thinspiration and fitspiration content, and found similarities with regards to messages that concerned body weight, thinness, eating guild and restriction, stigmatisation of fat, and objectification of the female form. Although these risky messages dominated the thinspiration content, they were also prevalent within the fitspiration group.

Whilst these findings provide great insight, Boepple and Thompson [25] analysed content featured on websites, rather than social media. Websites lack the interactivity provided by social media, where users can receive, construct, and communicate their ideas, instead of passively receiving content via a website. It is therefore likely that thinspiration and fitspiration content are presented differently on social media. Additionally, the researchers focussed upon the messages that the content conveyed, and did not specifically analyse and compare the bodies featured within this content.

There is a sparsity of research that has analysed and compared the physical attributes of thinspiration and fitspiration content, and to date there has been no research which has analysed bonespiration content. The present study will compare the bodies featured within thinspiration, bonespiration, and fitspiration to (i) examine the features of bonespiration content in relation to thinspiration and (ii) explore how fitspiration shared on social media compares to thinspiration and bonespiration. This research is important because previous studies have already associated exposure to this content with increased body dissatisfaction and decreased self-esteem, and it is therefore essential that the bodies featured within this content are more fully understood so that effective interventions and preventative measures can be developed.

## Method

### Sample

Images with the relevant hashtags for thinspiration, fitspiration, and bonespiration content were sampled from three social media platforms – Instagram, Twitter, and WeHeartIt. These platforms were chosen due to their popularity and their use of hashtag systems that facilitate photo-sharing [26]. Initially, 999 images were captured from 505 different social media profiles across the three platforms; however, whilst the authors appreciate there has been a female bias within research concerning body image, thinspiration and bonespiration appeared to feature more female bodies and so a decision was made to focus on images that contained only female bodies. After removing the images containing male bodies or no bodies at all, 734 images were analysed.

### Codebook

Prior to this study, no single unified codebook had been utilised across studies to analyse the bodies featured within the ‘inspirational’ content posted on social media. The codebook was derived from the previous codebooks employed by Ghaznavi and Taylor [19] in an analysis of thinspiration content, Deighton-Smith and Bell [24] in an analysis of fitspiration content, and Boepple and Thompson’s [25] comparison of thinspiration and fitspiration websites. The codes for content were also developed by the first and last author after an initial scoping review of the images which determined what codes were relevant for the analysis.

The bodies in the images were coded for body type and objectification. Body type was coded for to determine which body ideals were featured within the different types of content, codes for body type included: thin body, muscular body, bone protrusions, and presence of muscles. A thin body was operationalised as a body that was low in body fat, and a muscular body was defined as a toned body, bodies could be both thin and muscular [24]. Whilst there are many different types of bodies, the authors only coded for thin and muscular bodies as only these body types were observed in the initial scoping review of the images. In addition, these groups of content are supposed to be aspirational in nature and so it was likely that only these body types would feature in the imagery. Codes for bone protrusions included protruding ribs, collarbones, spines, and hips. These bone protrusions indicated the presence of an extremely thin body. The presence of muscles included codes for abdominal muscles and other muscles, which also informed the coding decision regarding the overall presence of a muscular body within the image.

The proportion of the body featured within the image was coded as an indicator of objectification of the body, which has been previously used as a code in other content analyses [19, 24]. An image that featured more than 50% of the body was coded as a full body, whilst an image that featured less than 50% of the body was coded as a half body. For this code, a decreased representation of the body indicated increased objectification, this is because decreased visibility of the body reduces the body to an object where it is seen in relation to its individual parts, and is not viewed in its entirety.

### Procedure

The images were sampled by inputting the hashtags ‘#thinspiration’, ‘#fitpiration’, and ‘#bonespiration’ into the search engines of Twitter, Instagram, and WeHeartit; however, as Instagram had banned all thinspiration content, adaptations were made to the hashtag language in order to locate this content. The website ‘Websta.me’ was used to identify alternative hashtags associated with the hashtags thinspiration, fitspiration, and bonespiration. Although bonespiration and fitspiration content were not restricted by blocking algorithms at the time of this study, alternative hashtags were also identified for these groups of content to maintain consistency, examples of these alternative hashtags included but were not limited to: ‘#thynspo’,’#thynspiration’,’#fitspo’,’#bonespooo’, etc. The top five alternative hashtags for each group were used each week to locate the sample and these hashtags were used across all three social media platforms to maintain consistency.

The sample of images was collected and archived using the data-archiving software Evernote across a period of six months between February 2015 and August 2015. The sample was then analysed using the codebook. During the analysis, the images were randomised so that the coding process was not impacted by exposure to the imagery, e.g. if the coder was exposed to only extremely thin bodies at one time, it was possible that they may view a thin body as larger than it appeared by comparison. A second coder analysed 10% of the sample to determine inter-rater reliability.

Ethical approval was acquired from the University of Bath Psychology ethics committee (Ethics Committee reference number = 15-017).

## Results

### Sample

The sample consisted of 734 images, 189 images with fitspiration tags, 269 images with thinspiration tags and 276 images with bonespiration tags. Chi-square tests were used to compare the prevalence of content between the thinspiration, fitspiration, and bonespiration groups.

### Interrater reliability

Cohen’s kappa was used to examine the interrater reliability of the codes. The Cohen’s kappa scores for the codes of content ranged from .724-.920, and thus exceeded the threshold score of .70 which is considered to be indicative of good reliability (Neuendorf, 2002). See Table 1 for a complete list of the kappa scores per variable.

**Table 1:** Percentages of images depicting body image attributes for each of the three different hashtags (fitspiration, thinspiration and bonespiration) and the overall chi square test and Cohen’s Kappa for each attribute

	Fitspiration	Thinspiration	Bonespiration	*χ2*	*p*	Cohen’s Kappa
*Body type*						
Thin body	81.1%^a^	95.4%^b^	94.2%^b^	43.6	<.001	0.844
Muscular body	46.2%^a^	9.4%^b^	4.0%^c^	165	<.001	0.857
*Muscles*						
Abdomen muscles	30.9%^a^	11.5%^b^	4.7%^c^	65.8	<.001	0.827
Other muscles	28.2%^a^	3.7%^b^	0.0%^c^	126	<.001	0.724
*Bone protrusions*						
Hip bones	9.0%^a^	22.8%^b^	26.4%^b^	22.1	<.001	0.920
Ribs	11.6%^a^	15.6%^a^	22.8%^b^	10.7	0.005	0.869
Collarbones	15.9%^a^	16.0%^a^	21.7%^a^	3.91	0.14	0.779
Spine	1.1%^a^	2.2%^a^	5.8%^b^	9.42	0.009	0.794
*Objectification*						
< 50% of the body shown	33.9%^a^	49.8%^b^	53.6%^b^	18.80	<.001	0.881
Full body shown	63.5%^a^	48.7%^b^	42.8%^b^	19.7	<.001	0.905

In each row, different letters indicate significant differences between these categories

### Body type

To understand what bodies are constructed and idealised within fitspiration, thinspiration, and bonespiration content, different body types were coded for. For this part of the analysis, a thin body was operationalized as a body that was low in body fat, and a muscular body was identified as body which was toned and featured muscles (Deighton-Smith & Bell, 2014). A body could be coded as both thin and muscular.

There was a significant effect of type of content on number of images containing a thin body, *χ* ^*2*^(2) = 32.05, *p* < .001. Pairwise comparisons showed that while thinspiration and bonespiration did not differ in terms of depicting thin bodies, *χ* ^*2*^(1) = 0.38, *p* = .54, thinspiration content did result in significant higher number of images containing thin bodies compared to the fitspiration content, *χ* ^*2*^(1) = 23.15, *p* < .001. A similar significant difference was found between bonespiration and fitspiration content, *χ* ^*2*^(1) = 19.13, *p* < .001.

In relation to the presence of muscular bodies, there was a significant effect of type of content on number of images containing a muscular body *χ* ^*2*^(2) = 156.39, *p* < .001. Fitspiration content featured a significantly greater proportion of muscular bodies, when compared to thinspiration content, *χ* ^*2*^(1) = 78.90, *p* < .001. In turn, the thinspiration content featured a significantly greater proportion of muscular bodies compared to the content of bonespiration images, *χ* ^*2*^(1) = 6.46, *p* = .011. This suggests that, compared to thinspiration and bonespiration content, fitspiration content may have less of a focus upon the thin ideal, and instead by comparison places a greater emphasis upon muscular physique than thinspiration and bonespiration.

In total, 7.94% of the fitspiration images, 5.43% of bonespiration images, and 4.09% thinspiration images were not coded as both thin and muscular. This indicates that the vast proportion of all three groups of content featured thin and muscular bodies.

### Muscles

The notion that fitspiration places a greater focus upon muscular bodies than thinspiration and bonespiration content was further supported when abdominal muscles and other muscles were considered. Fitspiration content contained significantly more abdomen muscles than the thinspiration content *χ* ^*2*^(1) = 26.36, *p* < .001. Likewise, the fitspiration content also featured a significantly greater amount of other muscles than the thinspiration content *χ* ^*2*^(1) = 55.27, *p* < .001. These differences were also found between the thinspiration and bonespiration content for the presence of abdominal muscles, *χ* ^*2*^(1) = 8.52 *p* = .004, and other muscles, *χ* ^*2*^(1) = 10.53, *p* = .001. Overall, fitspiration shows the highest level of muscles in the images, followed by thinspiration content, while bonespiration content had the lowest level of muscles visible in the analysed images.

### Bone protrusions

For this analysis, bone protrusions indicated the presence of an extremely thin body. A set of *χ* ^*2*^-tests showed significant differences across content groups for hip bones, *χ* ^*2*^(2) = 22.14, *p* < .001, ribs, *χ* ^*2*^(2) = 10.66, *p* = .005, and spines, *χ* ^*2*^(2) = 9.42, *p* = .009, but not for collarbones, *χ* ^*2*^(2) = 3.91, *p* = .14. When the presence of protruding bones was coded for across the groups of content, the previously observed difference between the thinspiration and fitspiration groups disappeared. As demonstrated in Table 1, the thinspiration and fitspiration group did not significantly differ across codes for protruding ribs, collarbones, and spines. A significant difference was only found between these groups on hip bone coding, with thinspiration content showing higher levels of hip bone visibility compared to the fitspiration content *χ* ^*2*^(1) = 14.98, *p* < .001. However, differences were found between the bonespiration and fitspiration groups in relation to bone protrusions, whereby the bonespiration group showed significantly larger prevalence of protruding hipbones, *χ* ^*2*^(1) = 21.90, *p* < .001, ribs, *χ* ^*2*^(1) = 9.40, *p* = .002 and spines, *χ* ^*2*^(1) = 6.77, *p* = .009. Compared to thinspiration, the bonespiration content showed significant higher visibility of ribs (*χ* ^*2*^(1) = 4.56, *p* = .03) and spines (*χ* ^*2*^(1) = 4.47, *p* = .03), but this was not the case for hip bones and collarbones.

This observed similarity between the thinspiration and fitspiration groups suggests that there might be a subgroup of fitspiration content, which is alike to thinspiration content, in relation to the idealisation of the extremely thin body; however, the prevalence of the extremely thin body within the fitspiration group did not equal the prevalence of this type of content featured within the bonespiration group.

### Objectification

In previous studies (e.g. Boepple & Thompson, 2015, Deighton-Smith & Bell, 2016), decreased visibility of the whole body has been associated with increased objectification of the body. In the present study, bodies were coded as either a half body (less than 50% of the body was visible) or a full body (more than 50% of the body was visible). It was therefore assumed that a greater prevalence of half bodies reflected an increased objectification of the body.

The analysis showed that the thinspiration group contained significantly more half bodies than the fitspiration group *χ* ^*2*^(1) = 11.51, *p* = .001. A similar difference was found when the bonespiration and fitspiration group were compared, whereby the bonespiration group featured a significantly greater proportion of half bodies than the fitspiration group *χ* ^*2*^(1) = 17.66, *p* < .001. The thinspiration and bonespiration groups did not differ significantly on this factor *χ* ^*2*^(1) = 0.79, *p* < .37. These findings suggest that fitspiration groups differed significantly from bonespiration and thinspiration content with regards to the objectification of the female body, whereby the thinspiration and bonespiration groups had a greater focus upon the objectification of the female form. Table 1 presents an overview of the findings.

## Discussion

The results from this study indicate that there are consistent differences between fitspiration content, when compared to thinspiration and bonespiration. Thinspiration and bonespiration content featured more thin objectified bodies; however, fitspiration content was similar to thinspiration content across codes for bone protrusions and, therefore, showed similarities with regards to the idealisation of the extremely thin body.

Similarities were generally observed between thinspiration and bonespiration content, yet bonespiration content contained fewer muscles and more bone protrusions which indicates that bonespiration may be a more extreme form of thinspiration that idealises the extremely thin body type.

The observed similarities between thinspiration and fitspiration content with regards to the presence of bone protrusions suggests that there is a subgroup of fitspiration content which is akin to thinspiration with regards to the idealisation of the extremely thin body type. This subsample of fitspiration content could be potentially dangerous due to the popularity and greater acceptance of this content. The everyday user could, therefore, be at risk of viewing this potentially harmful content that idealises the extremely thin female body.

The findings from this study should not be used to suggest that fitspiration is a healthy form of content or that the bodies featured within this content are ‘normal’ bodies. For example, when analysed in isolation, over 80% of the fitspiration imagery contained thin bodies and this figure only reached significance when compared to the other two groups of content. Also, almost one third of the fitspiration content featured abdominal muscles, which is not representative of the general population, and therefore suggests that there is an overrepresentation of abdominal musculature within fitspiration content. This can also be discussed in relation to thin body type idealised by thinspiration as a female would need to have low body weight for her abdominal muscles to be visible. So, whilst fitspiration contains less thin, extremely thin, and objectified bodies, the bodies featured within this content should not be seen as normal and it should still be approached with caution.

The present study adds to the current body of literature by further developing understandings of what body ideals are constructed on social media. This study has provided insight by utilising an observational method in a naturalistic setting, and due to the pictorial nature of the research offers visual insight into what female body types are idealised by users.

This study has replicated the findings of Ghaznavi and Taylor [19] as a large proportion of thin objectified bodies were identified within the thinspiration group. Although a less pronounced effect was found for bone protrusions within the thinspiration group, this could have been a product of the different platforms utilised for analysis. Likewise, the present study reflects Deighton-Smith and Bell’s [24] findings where a large proportion of the fitspiration content was coded as thin body (i.e. low in body fat) and muscular.

The present study adds to this body of literature by further developing these studies and conducting a direct comparison between the groups of content to highlight that fitspiration content does not as greatly impose thin and objectified bodies upon users, compared to thinspiration and bonespiration content.

The present study is the first to date to include bonespiration as a group for analysis. This adds to the understanding of what body types are constructed within the proED community on social media through the sharing of imagery, in which bonespiration content idealised a more extremely thin body type through the presentation of various bone protrusions. The bonespiration content also featured more objectified bodies compared to fitspiration content. This study, therefore, marks a baseline for further research to investigate bonespiration content.

Since the thinspiration and bonespiration groups contained such large proportions of thin and objectified female bodies, it raises important policy making questions about whether this content should be removed and blocked on social media, especially since this content is so easy to locate. This is a particular concern for the bonespiration content, which idealised extremely thin and more unhealthy body types. Despite this, as evidenced by the methodology utilised for this study, blocking this content would be an unrealistic and ineffective strategy for tackling this phenomenon. This is because social media users are easily able to manipulate the language of hashtags to sustain content and avoid the blocking algorithms. So, simply blocking content like thinspiration and bonespiration will not stop it from existing.

Instead of focusing on removing thinspiration and bonespiration, a more sustainable solution would involve developing interventions to minimise the impact of exposure to this content. One method could be to develop young people’s resilience towards this type of content using media-literacy programs [27]. By further investigating and developing understandings of thinspiration, fitspiration, and bonespiration, effective interventions and preventative measures can be informed, established, and implemented.

The present study also has implications for clinical practice particularly with regards to the treatment of individuals with eating disorders, such as anorexia or bulimia. Clinicians and parents need to be made aware of how easily bonespiration and thinspiration can be accessed on social media, and how actively engaging with this content could have negative consequences for an individual’s body image.

### Limitations

As this study was a content analysis, it could be argued that it was merely descriptive; however, descriptive studies of content hold a key place within communication research [28] and the present study marks a necessary step towards understanding the physical attributes of thinspiration, fitspiration, and bonespiration.

Furthermore, as the method incorporated adaptations of language to locate the thinspiration content, it is possible that the thinspiration sample represented an extreme form of thinspiration, whereby users actively used these alternative hashtags to ensure that the content was seen and sustained. Further research could explore this idea by investigating whether there are differences between content with similar hashtags such as ‘#thynspiration’ and ‘#thynspo’. This might explain some of the observed similarities between thinspiration and bonespiration content.

### Future research

To investigate these groups of content further, future research could adopt qualitative methods to gain a deeper insight into meanings that users construct by posting this content on social media. For example, future research could analyse the text that accompanies the imagery as well as the comments from other users.

Future research should also examine content that directly opposes the traditional thin ideal. For example, hashtags such as ‘#curvespo’ and ‘#thick’ are associated with a more voluptuous female body ideal and could be analysed and compared to the bodies featured within thinspiration, fitspiration, and bonespiration content.

Finally, future research could involve other social media platforms. Different platforms may attract different groups of users as shown by the diverse age demographics of Twitter, Instagram, and We Heart It [29]. A similar study by Ghaznavi and Taylor^19^ found that thinspiration content on Pinterest was less objectifying and placed a greater emphasis upon the muscular body, when compared to Twitter. Future research should account for this by segmenting the analysis by social media platform.

## Conclusion

In conclusion, fitspiration contained less thin and objectified bodies when compared to thinspiration and bonespiration. Similarities were found between thinspiration and bonespiration content, but bonespiration contained fewer muscles and more bone protrustions which indicates that bonespiration may represent an exaggerated form of thinspiration. The findings suggest that fitspiration content generally offers a less unhealthy body ideal alternative to thinspiration and bonespiration; however, there was a subgroup of the fitspiration sample which was alike to thinspiration with regards to the idealisation of the extremely thin body type, and as such users should still approach this content with caution. Future research should utilise qualitative methods to further develop understandings of the body ideals constructed within these groups of content and the motivations behind posting this content.
